# The Novel Introduction of a Thermal Camera in the Retrieval of a Retained Rectus Sheath Catheter: A Case Study

**DOI:** 10.7759/cureus.86892

**Published:** 2025-06-27

**Authors:** Mahmoud Awaly, Melanie Norton, Doaa Ahmed, Ahmed Abdelbar, Jessica Lim, D Al-Karawi, Shakir AL-Zaidi, Ahmad Sayasneh

**Affiliations:** 1 Gynaecological Oncology, Guy's and St Thomas' NHS Foundation Trust, London, GBR; 2 Obstetrics and Gynaecology, Guy's and St Thomas' NHS Foundation Trust, London, GBR; 3 Computer Science, Medical Analytica Ltd, Ewloe, GBR; 4 School of Life Course Sciences, Faculty of Life Sciences and Medicine, King's College London, London, GBR

**Keywords:** gynae oncology, rectus sheath catheter, retained surgical items, thermal imaging camera, variation in temperature

## Abstract

Midline laparotomy remains essential in gynecological oncology, and rectus sheath catheters (RSCs) are commonly used for postoperative analgesia. We report the case of a 46-year-old woman who underwent midline laparotomy for a suspected ovarian mass, which intraoperatively revealed mucinous ascites consistent with pseudomyxoma peritonei. An RSC was placed for pain control; however, during removal, a 5 cm segment remained embedded in the subrectus sheath. Initial imaging with plain X-ray failed to detect the retained catheter, which was eventually confirmed by computed tomography (CT). Intraoperative localization attempts using C-arm fluoroscopy and palpation were unsuccessful. A novel adjunctive approach using a Flir One Pro smartphone-based thermal camera (Flir One Pro Systems, Sweden), approved by clinical governance and operated by a trained surgeon, successfully identified localized heat on the abdominal wall, corresponding to inflammation caused by the retained catheter. Guided by this finding, a 2 cm incision was made to retrieve the coiled catheter without complications. The patient recovered well and was discharged on the third postoperative day. Histopathology confirmed pseudomyxoma peritonei, and the case was referred to a colorectal multidisciplinary team. This report highlights the feasibility and clinical value of thermal imaging as a radiation-free, real-time adjunct for detecting retained surgical materials, particularly when conventional imaging fails. Thermal imaging offers portability, precision, and safety, though its broader adoption requires standardized training, validation, and further research.

## Introduction

Despite advancements in minimally invasive abdominal surgical techniques, midline laparotomy remains essential for various elective and emergency procedures in gynecological oncology. Adequate post-laparotomy analgesia is crucial for patient comfort and early mobilization, which are key to reducing postoperative complications [1,2].

Rectus sheath catheters (RSCs) are commonly applied in major gynecological oncology surgeries to support postoperative recovery [3,4]. While usually reliable, RSCs may occasionally break, leaving retained fragments that require further investigation.

Imaging plays a critical role in identifying retained foreign bodies in surgical patients [5,6]. More common imaging modalities include X-ray (XR), computed tomography (CT), and ultrasonography (US). However, these methods have limitations, such as exposure to ionizing radiation (CT and XR), prolonged processing time, and reliance on radiological interpretation (CT, XR, US) [7].

There is limited literature on applying thermal imaging for diagnosing retained surgical instruments or its utility in intraoperative scenarios [8-10]. This novel application could reduce diagnostic delays, minimize unnecessary radiation exposure, and assist surgeons in retrieving retained instruments efficiently.

This case report aims to evaluate the feasibility and potential of infrared thermal imaging in gynecological oncological surgeries, particularly for retrieving retained surgical instruments.

## Case presentation

A 46-year-old woman presented with a 10-month history of abdominal distension, weight gain, breathlessness, and back pain. Imaging studies identified a complex ovarian cyst, necessitating further evaluation and surgical intervention. Her medical history included essential hypertension, hypertensive heart disease, type 2 diabetes mellitus, and undifferentiated inflammatory arthritis. The patient underwent a midline laparotomy performed by another member of the surgical team for the suspected ovarian mass. During the procedure, mucinous ascites and gelatinous matter in the abdomen suggested the presence of pseudomyxoma peritonei. Biopsies were taken from the mass and the peritoneal wall. Upon closure of the abdominal wall, an RSC was inserted to provide effective postoperative pain management.

**Figure 1:**
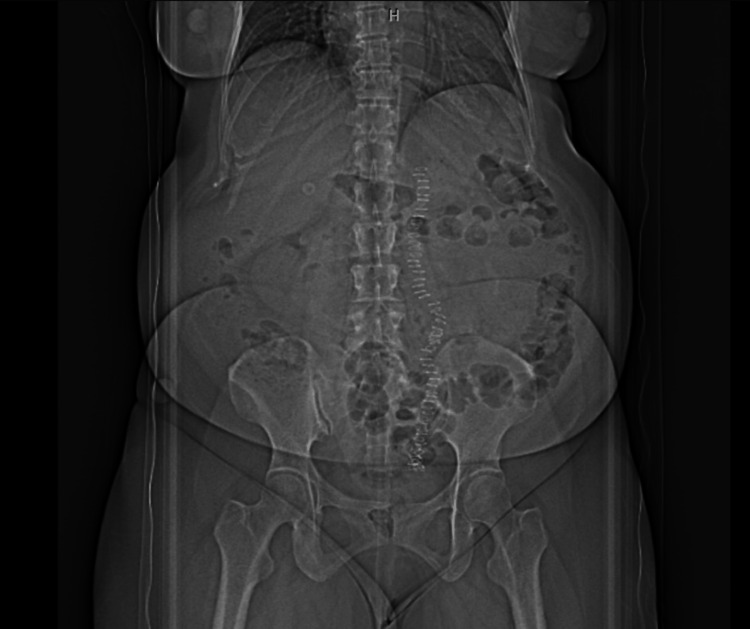
Preoperative X-ray showing the abdominal region Although radiopaque, the retained rectus sheath catheter fragment was small and difficult to identify definitively using this modality.

**Figure 2:**
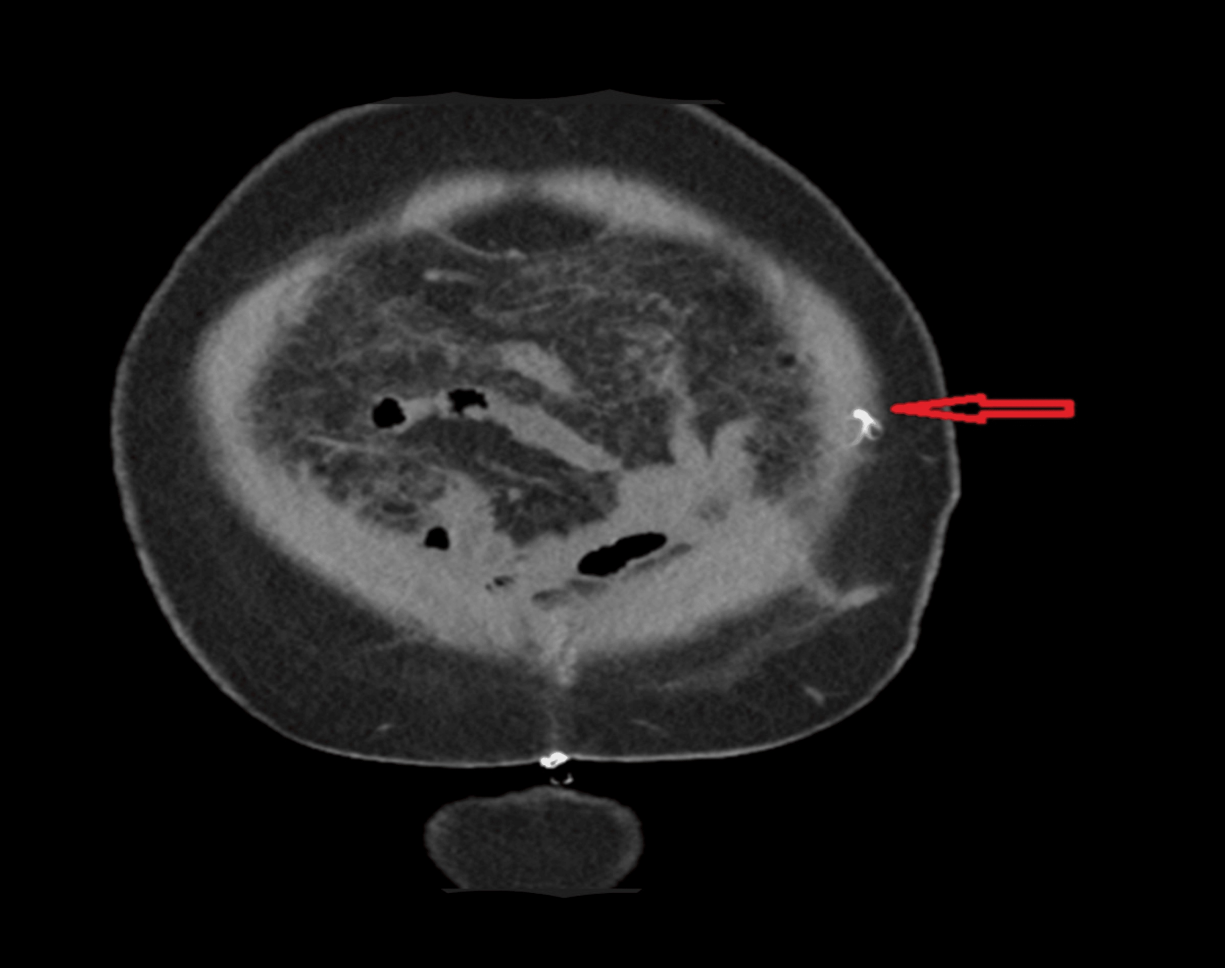
Preoperative CT scan Coronal CT scan image indicating the retained rectus sheath catheter fragment (highlighted by the arrow). Despite its radiopaque nature, the fragment’s small size presented challenges in definitive localization.

**Figure 3:**
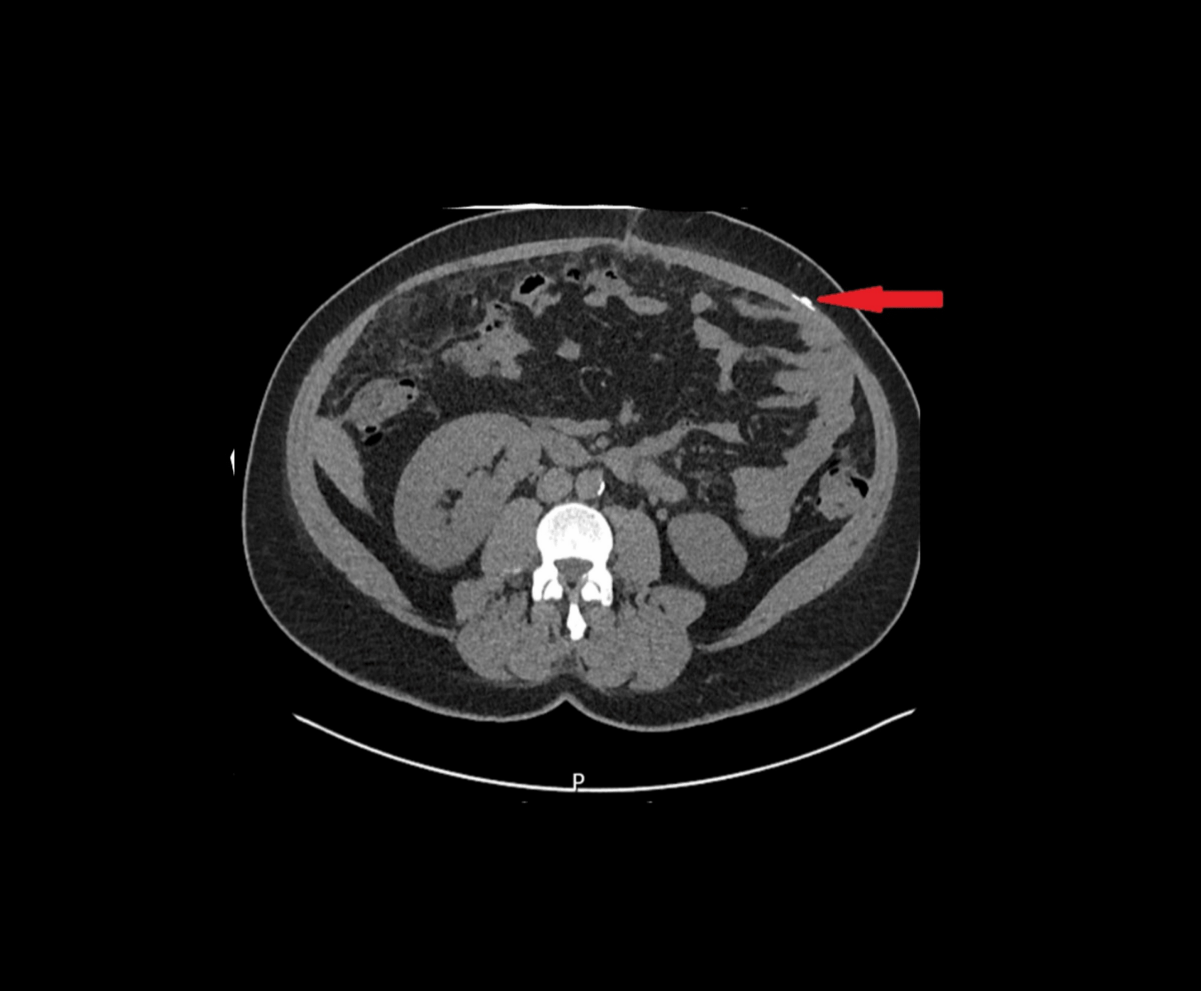
Transverse CT scan image showcasing the retained rectus sheath catheter fragment (highlighted by the arrow)

Postoperatively, during an attempt to remove the RSC by the same surgical team, a portion remained embedded, necessitating further intervention. A segment of the catheter, measuring approximately 5 cm, was entangled and coiled within the subrectus sheath. The retained piece was not visible in the plain abdominal XR (Figure 1). However, it was confirmed on CT scan imaging (Figures 2, 3) before the patient returned to the operating theater. It is noteworthy that the initial CT report showed no convincing evidence of the retained catheter. However, a further review by the main surgeon and a follow-up discussion with the radiology team confirmed the presence of the retained catheter. Thermal imaging was later used as an adjunct to pinpoint its exact location intraoperatively.

The patient provided written informed consent, including permission for anonymized photography and infrared thermal images during the surgical procedure. The lead surgeon introduced a thermal camera intraoperatively and captured thermal images using a Flir One Pro smartphone thermal camera (Flir One Pro Systems, Sweden). This portable device operates within the electromagnetic spectrum, detecting heat signatures ranging from 7.5 to 14 micrometers. The images were initially stored in an NHS iPad before storage in the hospital portal system (Epic Systems Corporation, 2024, US patents).

To ensure proficiency, Dr. Ahmad Sayasneh (AS) completed online training provided by the Infrared Training Centre (ITC) [10], which included introductory videos and articles. Additionally, AS obtained infrared thermography certification by completing a comprehensive three-day online course and passing an assessment conducted by Teletherm Infrared Systems/Research, Tampa, FL, USA [10]. Approval for the use of the Flir One Pro smartphone thermal camera (Flir One Pro Systems, Sweden) was granted by the Trust Department of Medical Physics. Further endorsements were obtained from the Trust’s Medical Photography Department and Clinical Governance. A service evaluation project was also approved by the Trust and assigned an official ID number.

**Figure 4:**
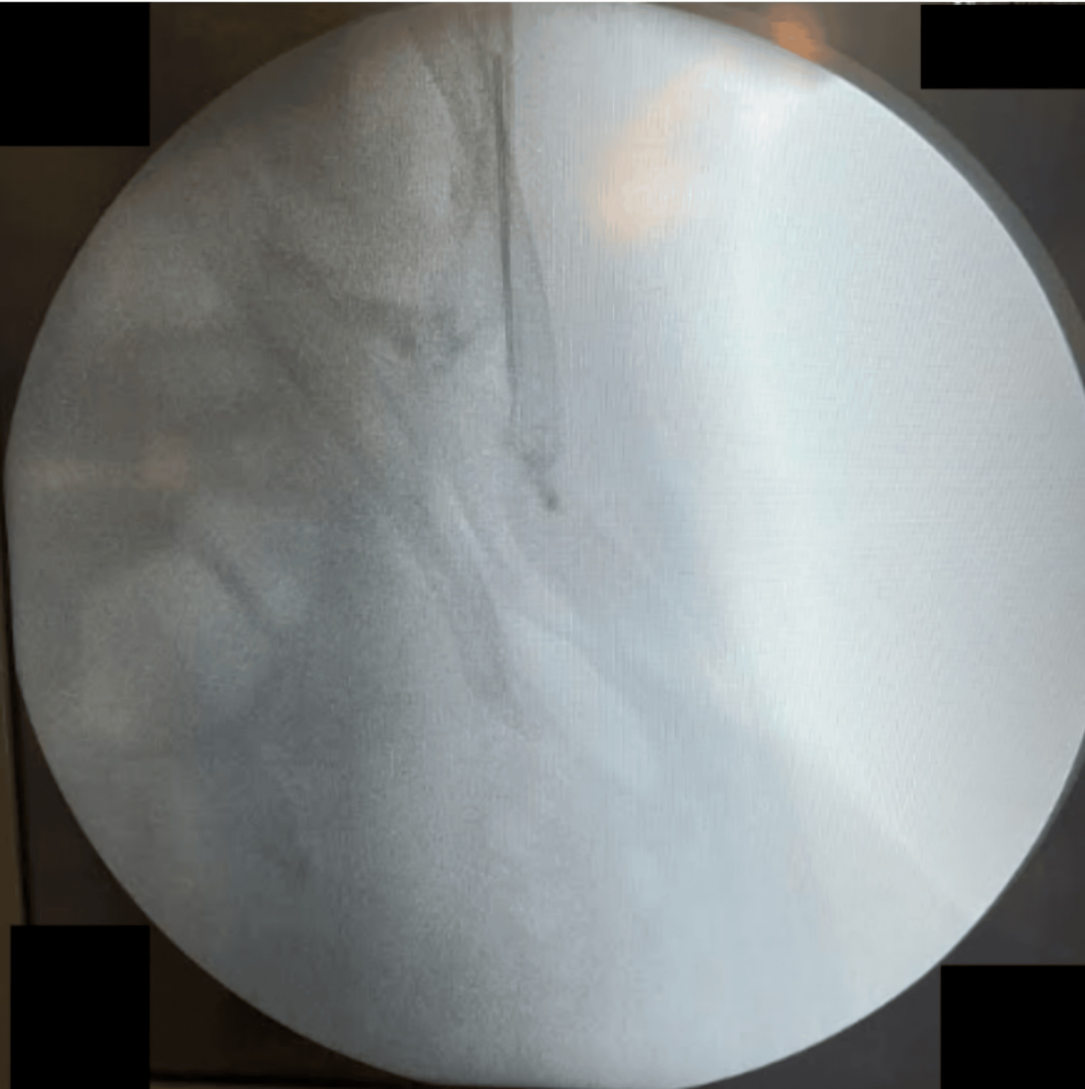
Intraoperative fluoroscopy, using a C-arm device, to localize the retained rectus sheath catheter fragment Despite its radiopaque nature, the small size of the fragment limited the efficacy of this imaging technique.

At first, the surgical team tried using a C-arm (portable imaging device), which uses XR imaging, to locate the lost part of the catheter, but was unsuccessful. XR imaging was less helpful in viewing the catheter (Figure 4). Clinical palpation failed to identify the catheter. There was no change in skin color on inspection. The patient provided written informed consent, including permission for anonymized photography and infrared thermal images during the surgical procedure. The lead surgeon introduced a thermal camera intraoperatively. Thermal images were captured using a Flir One Pro smartphone thermal camera (Flir One Pro Systems, Sweden). This portable device operates within the electromagnetic spectrum, detecting heat signatures ranging from 7.5 to 14 micrometers. The images were initially stored in an NHS iPad before storage in the hospital portal system (Epic Systems Corporation 2024, US patents).

**Figure 5:**
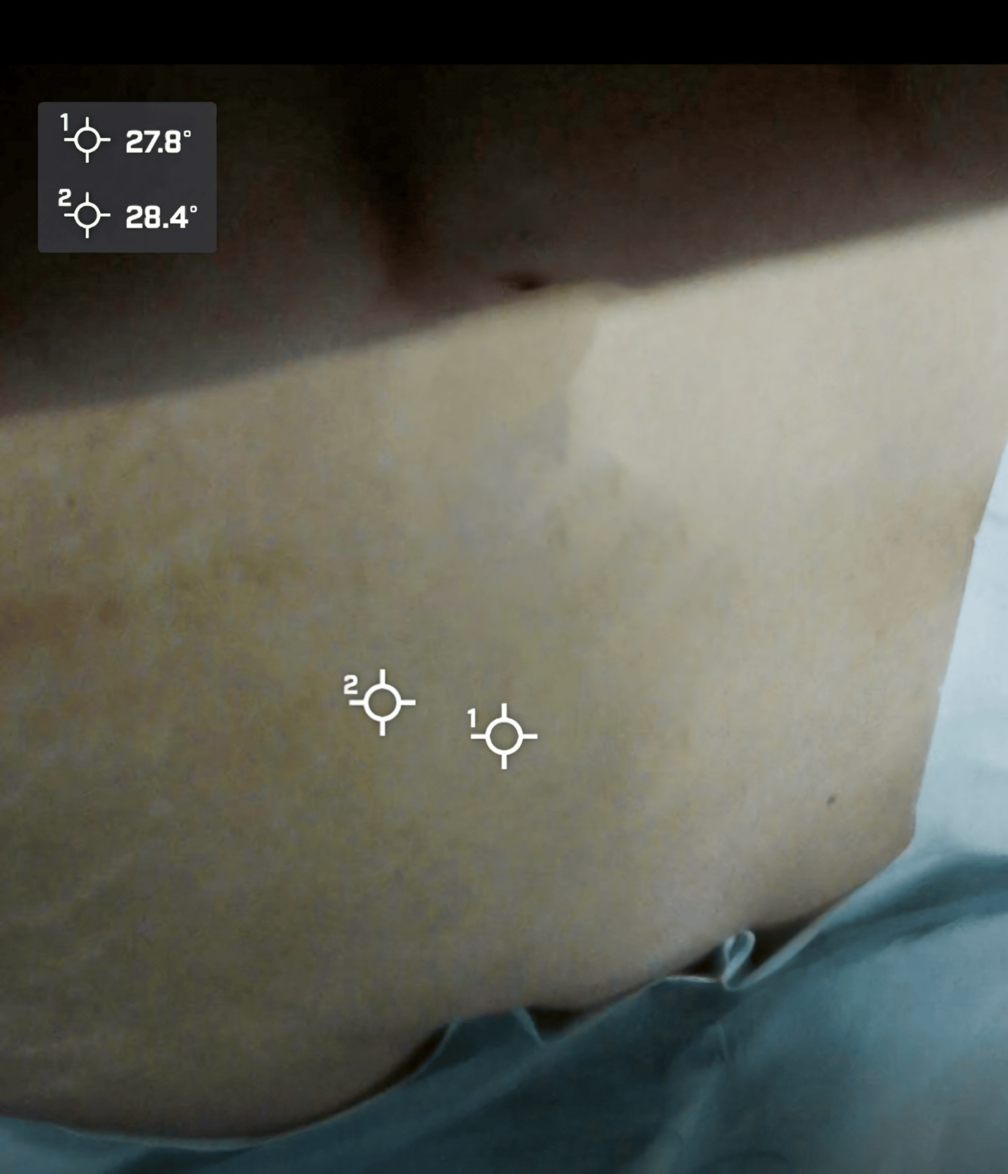
Intraoperative pre-incision thermal image Static digital image of the patient’s lower left abdomen, showing the area of interest with temperature markers (28.4°C and 27.8°C).

**Figure 6:**
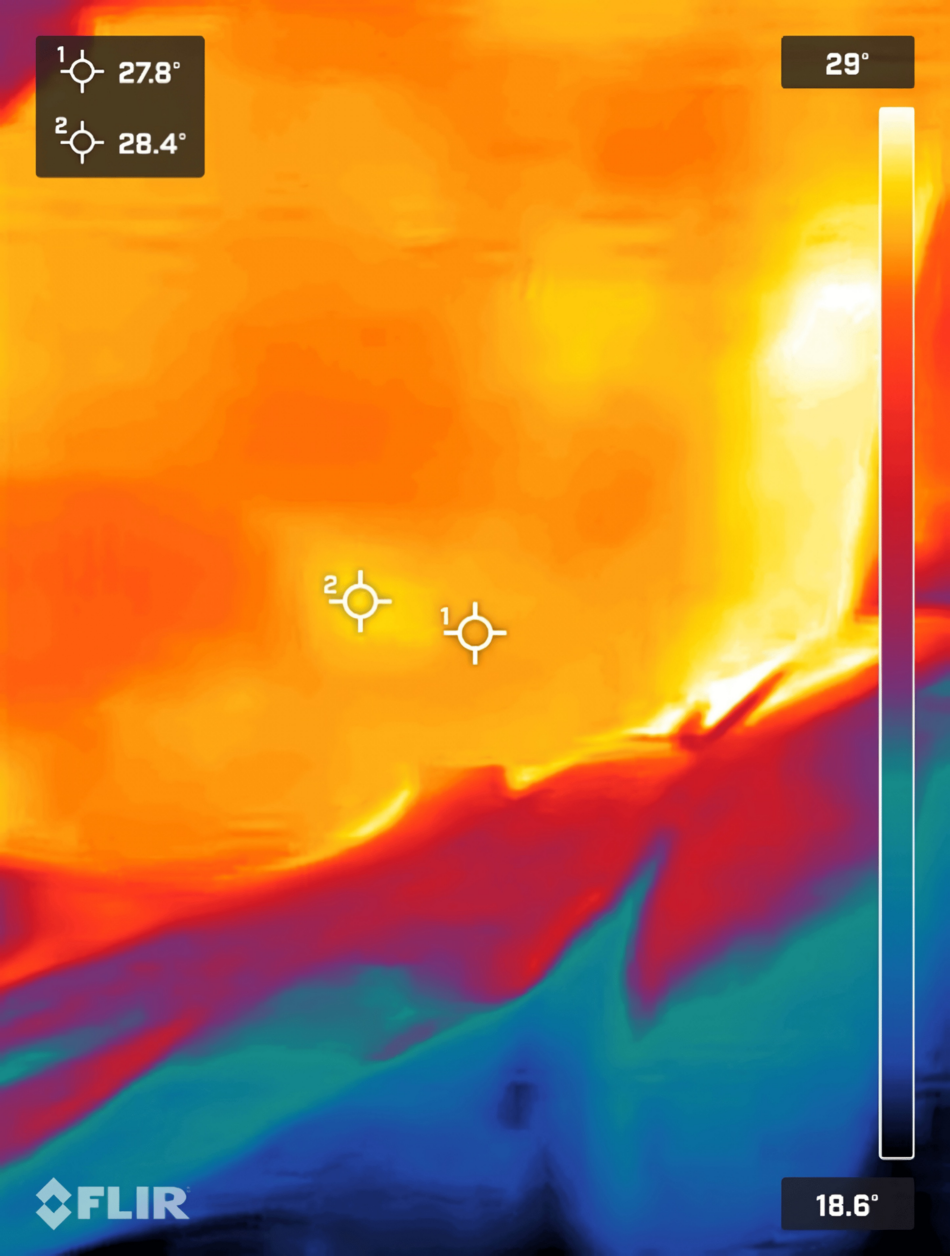
This corresponds to the site where thermal imaging was used to assist in retrieving the retained catheter fragment. Thermal images were captured using a Flir One Pro smartphone thermal camera (Flir One Pro Systems, Sweden).

**Figure 7:**
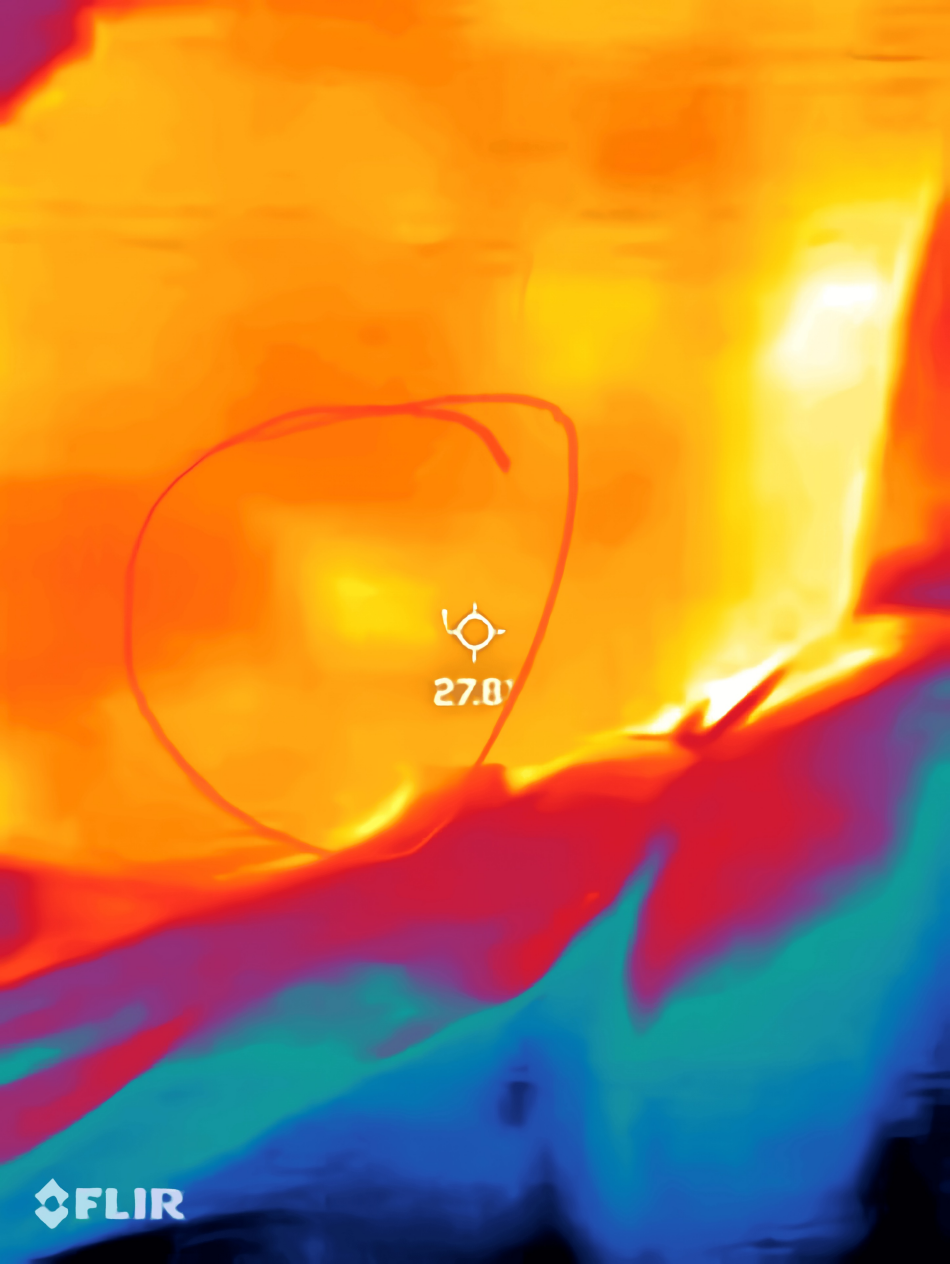
Same as Figure 6, with a circle drawn around the region of interest. Thermal images were captured using a Flir One Pro smartphone thermal camera (Flir One Pro Systems, Sweden).

**Figure 8:**
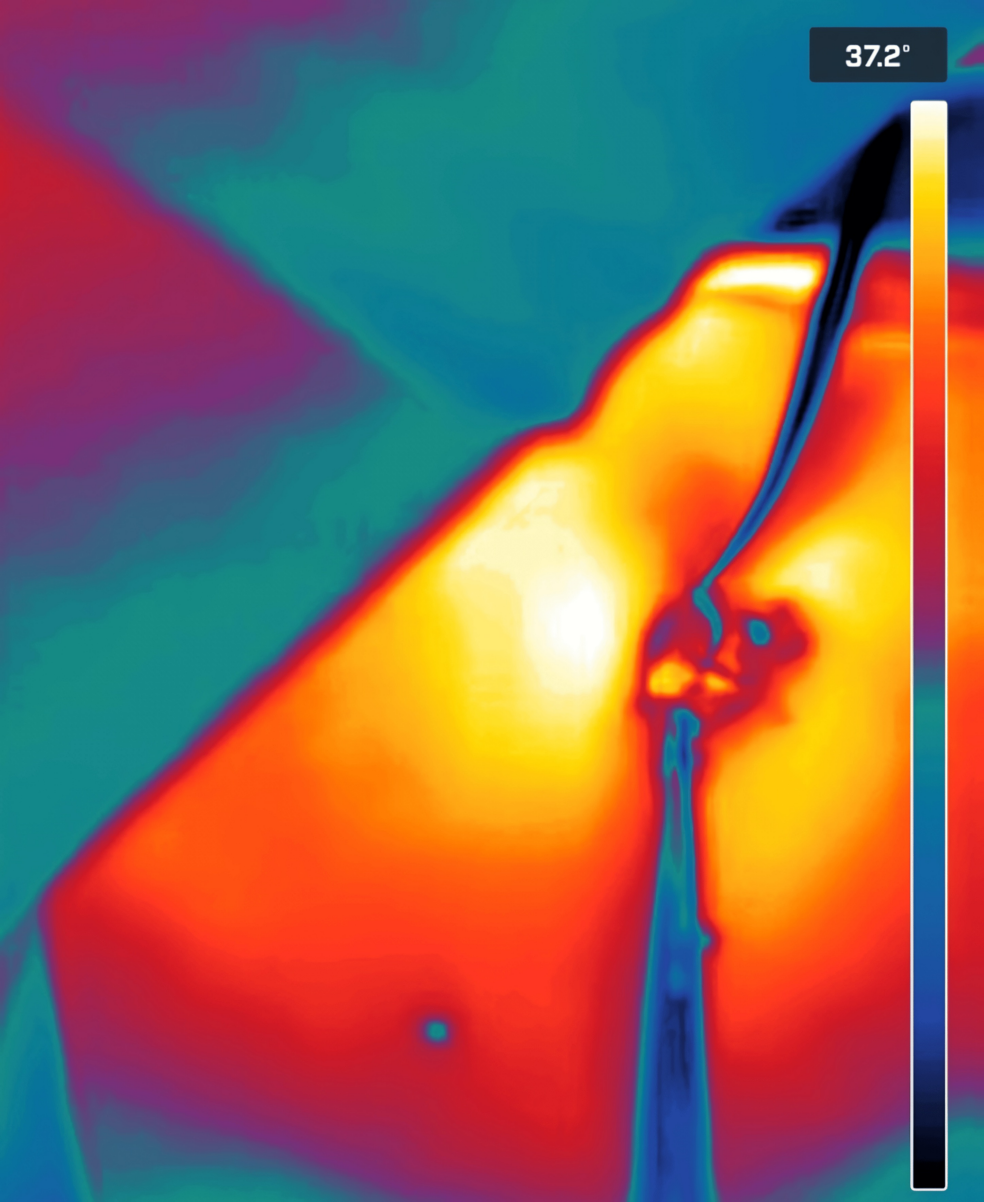
Enhanced thermal imaging of the surgical site, showing the cold color of the catheter (gray to black) compared to the surrounding tissue (yellow). The thermal color scale is on the right side of the image. Thermal images were captured using a Flir One Pro smartphone thermal camera (Flir One Pro Systems, Sweden).

**Figure 9:**
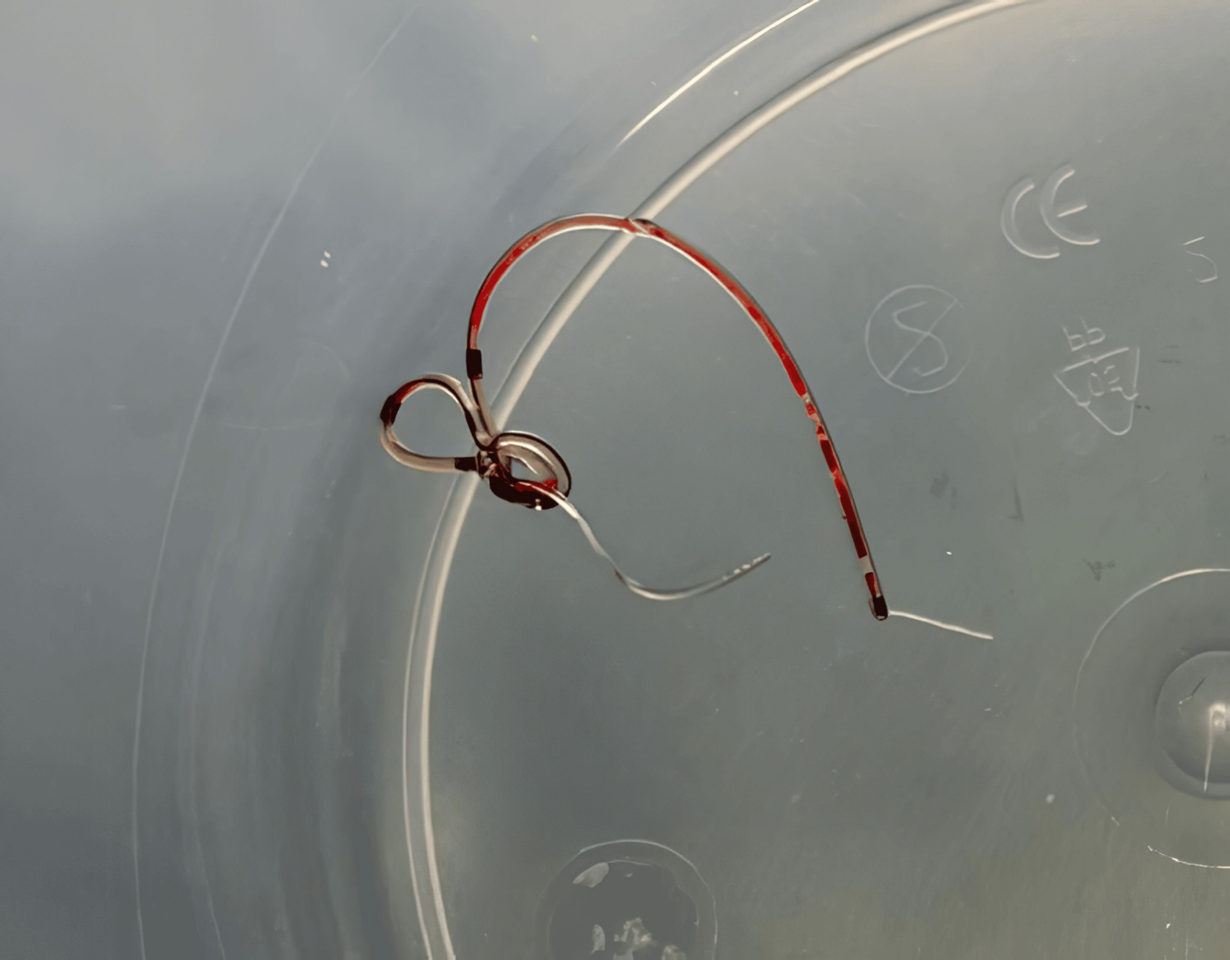
The rectus sheath catheter fragment retrieved after surgical removal, measuring approximately 5 cm. Note the coiled and entangled configuration of the fragment.

The thermal camera detected a slight increase in temperature on the patient’s left flank, indicating recent inflammation (Figure 5). Guided by thermal imaging, the surgical team made a 2 cm incision in the heated area. The thermal camera further facilitated the identification of the catheter fragment within the wound (Figures 6, 7), enabling its successful retrieval (Figures 8, 9). This approach minimized complications and optimized patient outcomes.

The patient recovered in the postoperative ward and was discharged three days later. Histopathological examination of the initial biopsies confirmed pseudomyxoma peritonei. Multidisciplinary team (MDT) discussions recommended referral to colorectal specialists for further evaluation and management.

The patient’s case was reviewed by a colorectal MDT, confirming the diagnosis of pseudomyxoma peritonei. A comprehensive management plan involving colorectal specialists emphasized the importance of timely and accurate diagnosis, multidisciplinary collaboration, and innovative techniques in complex oncological cases.

## Discussion

This case highlights the novel use of thermal imaging in surgical practice, specifically for retrieving a retained RSC in a patient with gynecological malignancy. Traditional imaging modalities, such as XR and CT, failed to conclusively identify the retained catheter due to the limitations of visualizing non-metallic materials. Thermal imaging provided a non-invasive, radiation-free alternative, enabling precise localization of the embedded fragment based on heat signatures indicative of inflammation.

The success of thermal imaging, in this case, underscores its potential as an adjunct tool in surgical practice. Thermal imaging offers several advantages, including real-time feedback, portability, and visualization of temperature changes associated with inflammation or tissue trauma. These benefits can minimize the need for additional invasive procedures, reduce time spent in the operating room, and improve overall patient outcomes.

Thermal imaging also holds promise for broader applications in gynecological oncology, including monitoring postoperative complications, such as infections or vascular compromise after skin flaps. However, its integration into routine clinical practice requires proper training and validation. In this case, the lead author underwent comprehensive training and certification, ensuring the safe and effective use of the technology.

Despite its advantages, thermal imaging has some limitations. It is sensitive to environmental conditions and operator expertise, and its interpretation can be subjective without standardized protocols [10]. Future research should focus on establishing clinical guidelines, validating their utility across various surgical contexts, and exploring their cost-effectiveness compared to traditional imaging modalities.

An unusual complication in using RSCs, such as the one presented in this case, is catheter knotting (Figure 5), as highlighted in a report by Doctor et al. [10]. Catheter knotting within the rectus sheath can complicate its use and retrieval and increase the risk of surgical reintervention. In our case, the coiled configuration of the catheter posed challenges in its localization and removal. 

## Conclusions

This case exemplifies the value of interdisciplinary collaboration and innovation in addressing complex surgical challenges. By incorporating advanced imaging technologies like thermal imaging as an adjunct to conventional methods, healthcare providers can complement diagnostic accuracy, refine surgical precision, and provide more effective care to patients with complex oncological conditions. 
